# Influence of Cultivation System and Proportion of Local Cultivars ‘Caaveiro’ and ‘Callobre’ in Flour Mixtures on the Nutritional Quality of Galician Bread

**DOI:** 10.3390/foods14101712

**Published:** 2025-05-13

**Authors:** M Pilar España-Fariñas, Joaquín Camba-Carrión, María Belén García-Gómez, María Lourdes Vázquez-Odériz, Matilde Lombardero-Fernández, Santiago Pereira-Lorenzo, Luis Urquijo-Zamora, Ángel Cobos, Olga Díaz, María Ángeles Romero-Rodríguez

**Affiliations:** 1 Areas of Nutrition and Food Science and Food Technology, Department of Analytical Chemistry, Nutrition and Food Science, Faculty of Sciences, Campus Terra, Universidade de Santiago de Compostela, 27002 Lugo, Spain; mariadelpilar.espana@rai.usc.es (M.P.E.-F.); joaquin.camba@rai.usc.es (J.C.-C.); mariabelen.garcia@usc.es (M.B.G.-G.); lourdes.vazquez@usc.es (M.L.V.-O.); angel.cobos@usc.es (Á.C.); olga.diaz.rubio@usc.es (O.D.); 2 Instituto de Biodiversidade Agraria e Desenvolvemento Rural (IBADER), Campus Terra, Universidade de Santiago de Compostela, 27002 Lugo, Spain; matilde.lombardero@usc.es (M.L.-F.);; 3 Agronomy and Animal Science Group, Department of Anatomy, Animal Production and Veterinary Clinical Sciences, Campus Terra, Universidade de Santiago de Compostela, 27002 Lugo, Spain; 4 Department of Crop Production and Engineering Projects, Escuela Politécnica Superior, Campus Terra, Universidade de Santiago de Compostela, 27002 Lugo, Spain; 5 Department of Crop Production, Agricultural Research Center of Mabegondo, 15318 A Coruña, Spain; luis.urquijo.zamora@xunta.gal

**Keywords:** bread quality, local cultivar, *Triticum aestivum*, farming system, organic wheat, nutritional profile, protected geographical indication

## Abstract

Bread is one of the main symbols of the culinary heritage of Galicia (NW Spain). This study evaluates the nutritional quality of Galician breads made from local wheat varieties, ‘Caaveiro’ and ‘Callobre’, under organic and conventional farming systems. Breads were prepared using 100% local wheat flour and a mixture of 25% local flour with 75% commercial flour, in accordance with the Protected Geographical Indication (PGI) ‘Pan Galego’. Nutritional composition was assessed using official AOAC procedures and validated enzymatic assays, including macronutrients, fiber, starch fractions, sugars and minerals. The results reveal that 100% local wheat breads showed significantly higher levels of protein, carbohydrates and minerals, which are beneficial for human health. Specifically, ‘Caaveiro’ breads were richer in protein, while ‘Callobre’ breads exhibited higher carbohydrate and mineral content. Although the cultivation system had a minor effect, it was still relevant when combined with the proportion of local flour. The study highlights the potential of local wheat varieties to enhance the nutritional value of Galician bread.

## 1. Introduction

Wheat bread has been one of the main staple foods for centuries, dating back to its first appearance around 14,000 BC in Jordan [1]. In Spain, bread is a fundamental part of popular culture and is deeply rooted in its traditions and social practices [2]. According to the latest data from the Ministry of Agriculture, Fisheries and Food, in its annual report on food consumption in Spain [3], Spanish households allocate 4.16% of their food spending to bread, which represents an average annual expenditure per capita of EUR 71.95 and an average annual consumption per capita of 27.35 kg. In Galicia (NW Spain), to preserve bread tradition, the Protected Geographical Indication (PGI) ‘Pan Galego’ has been recently registered [4], which certifies that Galician traditional bread is made from common wheat flour (*Triticum aestivum* L.), but a minimum of 25% of the flour must come from local wheat cultivars, such as ‘Caaveiro’ and ‘Callobre’, and following traditional methods, including sourdough fermentation. These practices contribute to its distinctive thick crust, tender crumb and slightly acidic flavor [5,6].

Wheat bread, which is typically made from wheat flour, water, yeast and/or sourdough starter and salt, is an important source of energy and offers a rich nutritional profile, including essential nutrients such as fiber, carbohydrates and proteins in the human diet [7,8,9].

In recent decades, there has been a growing interest in preserving and promoting high-quality bread made from local wheat varieties that maintain the authentic sensory qualities of Galician traditional bread, ultimately leading to the registration of the Protected Geographical Indication (PGI) ‘Pan Galego’. Based on studies conducted with local wheat varieties, the Mabegondo Agricultural Research Center (CIAM) selected and registered two soft wheat varieties based on their agronomic performance and grain quality for producing Galician bread. These varieties, ‘Callobre’ and ‘Caaveiro’, exhibit the characteristics of local ecotypes, as no crosses with foreign wheat varieties were used [10]. Furthermore, commercial varieties, when compared to local ones, show lower nutritional quality, with reduced content of minerals and other micronutrients [11] and less variability for protein content [12]. The better nutritional profile and sensory characteristics make traditional local varieties highly appreciated by consumers [13,14].

Moreover, traditional local varieties have strong adaptability to organic production conditions, due to their lower input requirements. Conventional production systems are associated with various environmental issues, such as the depletion of non-renewable energy resources, the reduction of biodiversity, water pollution, greenhouse gas emissions and the overuse of monocultures and tillage to obtain high yields [15]. Consequently, promoting this organic cultivation contributes to preserving and enhancing biodiversity, leading to environmental and agronomic advantages [16,17].

Thus, the nutritional importance of wheat bread and its influence on consumer perception and preference for organic products have sparked interest within the scientific community in recent years. Wang et al. [18] have studied the effect of the farming system on antioxidant activity, as well as phenolic and mineral content, considering two wheat species and two flour types. Similarly, there are other comparative studies between organic and conventional wheat flour at a nutritional level [19,20]. However, all these studies compared the flour rather than the final product, without accounting for the effect of processing on nutritional quality.

On the other hand, Fernández-Canto et al. [21] evaluated the nutritional quality of local Galician ‘Caaveiro’ wheat flours obtained through organic and conventional farming practices, as well as the bread produced from them. Despite research on the impact of cultivation systems on the nutritional quality of Galician bread, that study only focused on one of the two registered local Galician wheat varieties and did not consider the proportion of local flour incorporated into the dough used for bread making. In addition, the mineral content in Galician bread made with the ‘Caaveiro’ variety had been determined in different proportions (100% and 25%, mixed with commercial flour), but without considering the cultivation system [22]. To our knowledge, there is no comparative research that measures the nutritional composition of Galician bread made with both registered traditional varieties, considering the farming system and the proportion of local flour. For that reason, this study aimed to evaluate the effect on the nutritional composition of Galician bread between the ‘Caaveiro’ and ‘Callobre’ wheat varieties, taking into account the effects of the cultivation system (organic and conventional) and the proportion of local cultivar (100% and 25%, the minimum required content by the PGI ‘Pan Galego’).

## 2. Materials and Methods

### 2.1. Cultivation Conditions

To evaluate the influence of the wheat cultivation system and the proportion of local cultivars on the nutritional quality of Galician bread, the local (‘Caaveiro’ and ‘Callobre’) varieties of wheat (*Triticum aestivum* L.) were cultivated using both organic and conventional production systems. A completely randomized block design with 4 replicates was used. In total, 8 subplots were cultivated (2 types of cultivation × 4 repetitions). These plots were located at CIAM (Xunta de Galicia) (43°14′ N, 8°15′ W), in the northwestern of Spain. Each subplot was 10 × 20 m. The wheat was grown during the 2023 growing season.

For organic cultivation, broiler manure was applied at a rate of 8.5 t/ha, and weed control was managed using tine harrow passes. In conventional cultivation, a basal mineral fertilizer was applied at a rate of 350 kg/ha of a complex fertilizer (8-15-15), and a top-dressing mineral fertilizer was applied at a rate of 120 kg/ha of calcium ammonium nitrate (CAN 27%). During the tillering phase of the crop, a selective herbicide (40% chlortoluron + 2.5% diflufenican) was applied on the conventional strips of wheat in March, at a dosage of 2 L/ha for weed control. In May, an application of a fungicide, at a dosage of 1 L/ha (tebuconazole), was carried out to prevent and control the development of foliar diseases during the grain filling stage, in the conventional farming.

The grain from the local cultivars of Galicia was milled in stone mills by the company Grupo Da Cunha, Carral, A Coruña, Spain, while commercial flour, used for mixtures, was refined in industrial mills.

### 2.2. Bread Samples

Eight different types of bread loaves were prepared in duplicate at a traditional bakery (company Grupo Da Cunha, Carral, A Coruña, Spain), under supervision. They used 7 kg of wheat flour, 4.1 L of warm tap water, 2.5 L of sourdough starter, 70 g of commercial baker’s yeast (*Saccharomyces cerevisiae*) and 140 g of refined common commercial salt.

To evaluate the influence of the proportion of local flour in flour mixtures on the nutritional quality of Galician bread, bread was made with 100% of the two local varieties (‘Caaveiro’ and ‘Callobre’) and with the minimum required content by the PGI ‘Pan Galego’ (25% local wheat flours and 75% commercial flours) from both cultivation systems (organic and conventional).

Therefore, the wheat flour samples used are detailed in Table 1.

**Figure 1:**
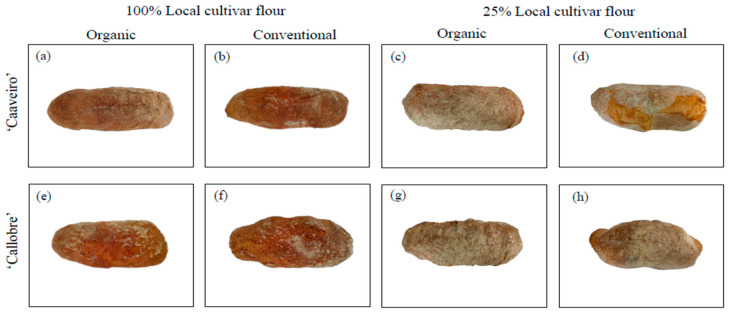
Bread samples analyzed. (**a**) ‘Caaveiro’, 100% local cultivar flour, organic farming; (**b**) ‘Caaveiro’, 100% local cultivar flour, conventional farming; (**c**) ‘Caaveiro’, 25% local cultivar flour, organic farming; (**d**) ‘Caaveiro’, 25% local cultivar flour, conventional farming; (**e**) ‘Callobre’, 100% local cultivar flour, organic farming; (**f**) ‘Callobre’, 100% local cultivar flour, conventional farming; (**g**) ‘Callobre’, 25% local cultivar flour, organic farming; (**h**) ‘Callobre’, 25% local cultivar flour, conventional farming.

All the bread samples (Figure 1), from both cultivation systems (organic and conventional), both local wheat varieties (‘Caaveiro’ and ‘Callobre’) and both proportions of local flour (100% and 25%), were made with the same baking procedures by the PGI ‘Pan Galego’ specifications [4]. The dough was kneaded and underwent bulk fermentation for 1 h without being divided. After fermentation, the dough was divided into smaller portions and shaped into balls. The doughballs were then left to rest for 20 min. Following the rest period, the dough was shaped as long, rustic loaves, characterized by a thick, crisp crust and a moist crumb, distinctive of traditional Galician bread. The loaves were then allowed to ferment for an additional 2 h. Two independent batches were prepared for each bread type, and all breads were baked in the same traditional stone oven for 1 h at a temperature of 220 °C.

### 2.3. Nutritional Analysis

The nutritional composition analysis of Galician bread was analyzed as described in AOAC methodology [23]. The analysis was performed in duplicate unless otherwise stated.

#### 2.3.1. Moisture

The moisture content was determined following AOAC method 925.10 [23], in which 3 g of the fresh bread sample, including both crust and crumb in a representative manner, was placed in a metal dish and weighed. The samples were then placed in an oven at 105 °C until a constant weight was achieved. Moisture content was determined by calculating the difference in weight. The moisture content was expressed as g/100 g of fresh weight.

#### 2.3.2. Crude Protein

The crude protein content was evaluated by the Kjeldahl method (N × 6.25), using the AOAC 920.87 method [23], using automatic distillation. The crude protein content was expressed as g/100 g of fresh weight.

#### 2.3.3. Ash

The ash content was calculated by incineration of 0.5 g of the sample at 550 ± 15 °C in a muffle, following the AOAC method 923.03 [23]. The ash content was expressed as g/100 g of fresh weight.

#### 2.3.4. Crude Fat

A Soxhlet apparatus was used to extract and measure the crude fat of a known weight of the powdered sample with petroleum ether and diethyl ether. The crude fat content was expressed as g/100 g of fresh weight.

#### 2.3.5. Carbohydrates

Carbohydrates were determined by difference, subtracting the respective percentages of moisture, protein, ash and fat from 100%. The carbohydrates content was expressed as g/100 g of fresh weight.

#### 2.3.6. Starch

The contents of rapidly digestible starch (RDS), slowly digestible starch (SDS), total digestible starch (TDS) and resistant starch (RS) were determined in bread samples with the digestible and resistant starch assay kit (K-DSTRS) (Megazyme International Ireland Ltd., Wicklow, Ireland). Total starch (TS) content was determined by the sum of TDS and RS. The analysis was performed on freeze-dried bread samples and the results were expressed as g/100 g of fresh weight.

#### 2.3.7. Simple Sugars

The glucose, fructose, maltose and sucrose contents of Galician bread were measured using an ion chromatograph (930 Compact IC Flex, Metrohm, Madrid, Spain) with a Metrosep Carb 2 250/4.0 column and Metrosep Carb 2 Guard/4.0 pre-column. Detection was performed amperometrically with a 945 Professional Detector Vario 1 at 30 °C, using a 300 mM NaOH/1 mM NaAc eluent at a flow rate of 0.5 mL/min, with an injection volume of 20 µL. The analysis was performed in quadruplicate and the simple sugar composition was expressed as g/100 g of fresh weight.

#### 2.3.8. Amylose and Amylopectin

The amylose and amylopectin contents were determined using the Megazyme Amylose/Amylopectin Assay kit K-AMYL (Megazyme International Ireland Ltd., Wicklow, Ireland). The analysis was performed on freeze-dried bread samples. The amylose and amylopectin contents were expressed as g/100 g of starch.

#### 2.3.9. Fiber

The content of total fiber in Galician bread was measured following the AOAC method 985.29 [23], using the Megazyme total dietary fiber analysis kit K-TDFR-100A (Megazyme International Ireland Ltd., Wicklow, Ireland). The analysis was performed on freeze-dried bread samples and total fiber content was expressed as g/100 g of fresh weight.

#### 2.3.10. Minerals

The macroelements (Ca, Mg, Na, K and P) and microelements (Cu, Fe, Mn, Zn and Se) of Galician breads were analyzed by an ICP-MS (Agilent 7700x, Agilent Technologies, Madrid, Spain) with a sample introduction system consisting of a Micromist glass low-flow nebulizer, a double-pass glass spray chamber with a Peltier system (2 °C) and a quartz torch. The analysis was performed in quintuplicate and the mineral composition was expressed as mg/100 g of fresh weight.

### 2.4. Statistical Analysis

The statistical treatment of the data was performed using IBM SPSS Statistics software, version 28.0 (IBM Corp., Armonk, NY, USA). Means and standard deviations were calculated. A three-factor analysis of variance (ANOVA) was used to study the effect of the factors (cultivation method, wheat variety and local wheat flour proportion) and their interactions, including both two-way and three-way interactions, as fixed sources of variation. When the interactions were significant for some physicochemical parameters, they were studied in more depth by segmenting the data matrix into different levels of cultivation systems, local wheat cultivar and local wheat flour proportion, and a Student’s *t*-test for independent samples was performed. The statistical significance level was set at *p* < 0.05. Principal component analysis (PCA) was conducted using the open-source software R (version 4.3.2) [24], specifically with the FactoMiner package (version 2.8.) [25] and the Factoextra package (version 1.0.7.).

## 3. Results

In terms of the chemical parameters analyzed, the descriptive statistics of nutritional profile of all analyzed breads (mean ± standard deviation) are displayed in Table 2 and the F-statistic and *p*-value obtained for the ANOVA (individual factors, two-way factor interaction and three-way factor interaction) are shown in Table 3.

**Figure 2:**
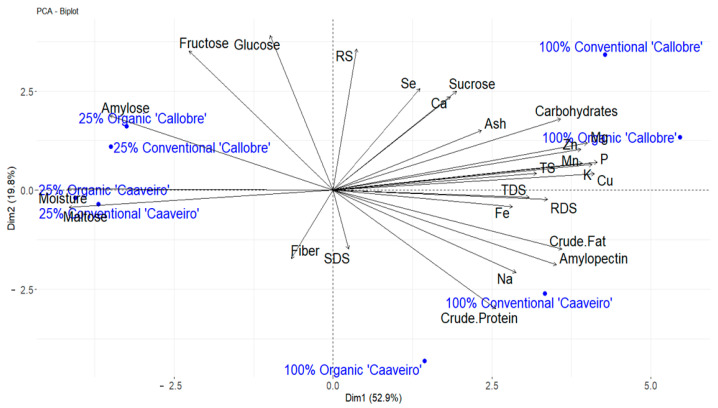
Principal component analysis (PCA) performed with chemical compositional data of the analyzed bread samples made with Galician wheats ‘Caaveiro’ and ‘Callobre’ (25 and 100% mixed with commercial flour) cultivated in organic and conventional systems.

PCA was used to explain differences among the Galician bread samples from a nutritional composition point of view (Figure 2). The first two dimensions of the analysis account for 72.69% of the total data variability, a high percentage that highlights the ability of these to capture a significant portion of the overall variation.

Among the factors evaluated, the cultivation method generally showed the least influence on the nutritional characteristics of the breads, as both the organic and conventional samples of the different breads appear grouped together. However, there was clear separation between the organic and conventional samples made with 100% ‘Caaveiro’ flour. This suggests that, under specific conditions, such as when local flour is used exclusively, the farming system may demonstrate a more pronounced effect on the bread’s nutritional profile. In the two-dimensional space, it was possible to observe the clearly differentiated behavior between the loaves of bread made with 100% ‘Caaveiro’ and ‘Callobre’ flours and those made with only 25% local flour. Considering the positions of the samples in the two components, differentiated behaviors were observed between the bread samples depending on their proportion of local cultivar flour in the dough used for bread making. The high proportion of foreign flour (75%) reduces the potential differences between the bread made with local flour. However, breads produced with 100% local flours, ‘Caaveiro’ and ‘Callobre’, are distinctly separated, reflecting the inherent differences between these two registered varieties. Therefore, the principal component analysis (PCA) allows for the differentiation of three groups: (1) breads made with 25% local flour; (2) breads made with 100% ‘Caaveiro’ local variety flour; and (3) breads made with 100% ‘Callobre’ flour.

Galician breads made with 25% local variety flour show high values for moisture, maltose and amylose. However, these breads exhibit low values for fat, P, Cu, Mn, K, Mg, Zn, protein and total digestible starch (TDS). Breads made with 100% ‘Caaveiro’ flour show high values for protein but low values for fructose and glucose. Finally, the group of breads made with 100% ‘Callobre’ flour is characterized by high values for carbohydrates, Zn, Mg, Mn, P, K and Cu, and these breads have low values for maltose and moisture.

## 4. Discussion

The proportion of local wheat flour had the greatest effect on the nutritional composition of the breads. It significantly influenced various components, including moisture, protein, fat, carbohydrates, fructose, amylose, amylopectin, magnesium (Mg), phosphorus (P), potassium (K), manganese (Mn), iron (Fe), copper (Cu) and zinc (Zn). The moisture content of bread is a strong indicator of its shelf life. Lower moisture is associated with better stability during storage [26]. The bread made with 100% Galician flour showed a lower moisture content than that of bread made with mixture flours. The moisture content of bread made with 25% of local wheat flour was similar to the results obtained in a study conducted by Carocho et al. [27] that compared different types of bread made with a base of wheat flour and another type of cereal in a certain percentage. Moreover, although the breads made with a local variety and commercial flour mixture, compared to those made with 100% local flours, had significantly higher glucose, fructose and maltose content, these values remain very low, as simple sugars serve as substrates for lactic acid bacteria and yeasts during fermentation [28]. However, breads made with 100% local flour generally showed higher nutritional values, indicating that increasing the proportion of local Galician wheat varieties enhances the nutritional quality of Galician bread. Thus, protein, fat and carbohydrates contents were higher when the bread was made entirely with 100% local wheat compared to those made with only 25% of it. Boukid et al. [29] studied some quality properties of flours from landraces, old and modern genotypes of Tunisia, and found that landraces also showed the highest protein content.

Furthermore, bread is an important source of energy in the human diet due to its high content of digestible starch [30]. The superior levels of total starch (TS) and total digestible starch (TDS) in bread made with 100% flour from the local cultivar ‘Caaveiro’, particularly under organic cultivation, could help meet daily human energy requirements, considering the recommendation from the Spanish Agency for Food Safety and Nutrition (AESAN) to obtain at least 50% of total dietary energy from carbohydrates [31]. In addition, AESAN recommends including starch-rich foods in each meal, as a complex carbohydrate that digests slowly, requiring enzyme action to break down into simple sugars [32].

The bread made with 100% local flour also showed a significantly higher content of macroelements and microelements, which are essential for the biochemical and physiological functions of the human body [33]. In previous research Fernández-Canto et al. [22] analyzed the element content of the flour and bread made with ‘Caaveiro’ and commercial flour, both individually and mixed. This work reported that breads made with ‘Caaveiro’ flour showed above average values for Cu and below average values for Ca and Se. Although in the present study bread made with 100% local wheat varieties also exhibited a higher level of Cu, the content of other minerals (Na, Mg, P, K, Ca, Mn, Fe, Cu and Zn) was also found to be superior compared to bread made with mixture flours. Specifically, 100% ‘Callobre’ breads generally had a higher content of several minerals (Mg, P, K, Ca, Mn, Cu and Zn) than 100% ‘Caaveiro’, which may explain the differences obtained compared to the previous work, in which only the ‘Caaveiro’ local variety was studied. Iron (Fe) and magnesium (Mg) help reduce tiredness and fatigue; manganese (Mn), copper (Cu), selenium (Se) and zinc (Zn) contribute to the protection of cells against oxidative stress; calcium (Ca) and magnesium (Mg) support normal muscle function; and phosphorus (P) helps maintain normal metabolism [34]. Thus, bread made with 100% flour from a local variety has proven to be a valuable source of micronutrients that contribute to good human health.

Lockyer and Spiro [35] determined that, on average, bread in the UK provides 11–12% of daily energy intake, 16–20% of carbohydrates and 10–12% of protein across all age groups and is a significant source of micronutrients, contributing 15–17% of iron, 12–17% of calcium, 12–13% of magnesium and 10–11% of zinc. Therefore, the higher concentrations of carbohydrates, proteins and essential minerals (Fe, Ca, Mg and Zn) found in Galician bread made with 100% local varieties further support its role in meeting the daily nutritional requirements of the human diet. In addition, the results previously reported by Valli et al. [36] demonstrate that breads made with ancient grains, in comparison with modern ones, offer benefits for human health.

On the other hand, the local wheat variety also played a significant role in shaping the nutritional profile of the breads. Breads made with 100% ‘Caaveiro’ wheat flour showed significantly higher levels of protein, fructose and glucose. In contrast, those produced with 100% flour from the local cultivar ‘Callobre’ were characterized by a higher content of amylose, carbohydrates, sucrose and minerals and a significantly lower content of maltose and moisture. Differences in mineral content have also been previously described among different genotypes, as cited in a review by Lammerts van Bueren et al. [37]. A recent study has compared the physicochemical composition and the consumer perception of Italian breads made with seven common Italian wheat varieties and a new cultivar with high bread-making quality, ‘Aquilante’ [38]. Their results suggest that old-cultivar-based bread differed from the new one and gave the most appreciated bread. Similarly, Melini et al. [39] have measured the nutritional composition of ‘Pane di Monreale’, a traditional Italian bread, by using two durum wheat local varieties, ‘Russello’ and ‘Tumminia’. Some differences were observed: the bread made with the ‘Russello’ variety showed a higher crude fat content, in contrast to the one made with ‘Tumminia’ wheat, which had a significantly higher available starch content.

Regarding dietary fiber content, total fiber levels were generally higher in bread made with 100% local flour compared to those with 25% local flour. This effect was more evident in breads made from ‘Callobre’ wheat. Although differences between farming systems were not statistically significant, there was a tendency for bread made with organic flour to show higher total fiber values, which is consistent with previous findings reported by Fernández-Canto et al. [21]. In this context, only total dietary fiber was determined in the present study. This decision was based on the aim to provide a global assessment of the bread’s nutritional quality, considering that total fiber is the value most commonly used in food labeling and nutritional claims.

The cultivation system, organic or conventional, was found to be the least influential factor in this study. It only significantly affected the content of certain components such as Na, iron (Fe), magnesium (Mg), manganese (Mn), zinc (Zn) and copper (Cu) in the bread samples. Organic production typically has some influence on the mineral content due to differences in fertilizer used [40,41,42], but, in this case, its overall impact on the nutritional characteristics of the Galician breads was minimal. Bread made with organic wheat flour showed higher levels of Na, Mn and Zn but lower levels of Mg, Fe and Cu compared to conventional wheat bread. A significantly lower iron content and higher zinc levels in organic wheat flour were previously reported by Vrček et al. [43]. However, in this study, Mn content was lower in flour from organic wheat. Moreover, in the results reported by Fernández-Canto et al. [44], bread made with organic wheat flour demonstrated higher Na levels, as observed in this study, but also higher Fe and Cu levels, which differ from our findings. These discrepancies across the literature suggest that multiple factors such as soil composition, fertilizer inputs, environmental conditions, geographical location or post-harvest processes can significantly influence mineral retention [45].

Regarding the influence of the cultivation system on the protein content, there does not appear to be a clear trend. This is because the breads made with organic wheat flour of the ‘Caaveiro’ variety have a higher protein content than conventional ones; however, when comparing the breads made with ‘Callobre’ flour, this trend does not hold. There is also no clear trend in the bread made with different proportions of local wheats. Fernández-Canto et al. [21] evaluated the protein content of the ‘Caaveiro’ wheat flour variety based on the cultivation system and no significant differences were observed, suggesting that the same level of protein can be achieved in the flours using organic practices. Based on this, it cannot be concluded that the cultivation system has a clear effect on protein content, although some authors suggest that protein content may be improved by crop management practices, especially through nitrogen fertilization levels [46].

A direct comparison with the study by Fernández-Canto et al. [21] reveals both convergences and divergences. While both studies confirm the influence of the cultivation system on certain nutritional parameters, the absolute values differ for several variables, including ash, Fe and Ca. These discrepancies may stem from differences in environmental conditions across growing seasons and flour milling processes. Furthermore, the scope of the present work extends beyond that of Fernández-Canto et al., as it includes a second local variety (‘Callobre’) and incorporates the analysis of different flour incorporation levels (25% and 100%), allowing for a deeper understanding of the interactive effects of genotype, cultivation system and formulation.

All these results suggest that the cultivation system is not a major determinant in defining the broader nutritional profile of the breads. In agreement with our findings, many studies also found no significant differences between the two cultivation systems in terms of concentration of various quality parameters in wheat bran and refined flour [47] and in wheat ears and grains [48]. However, as already mentioned, organic farming conditions offer numerous environmental and agronomic benefits [49]. Therefore, promotion of organic production is recommended.

The interactions between cultivation systems and the proportion of local flour or the local wheat variety suggest that, whereas the cultivation method alone has a limited effect, it can become more relevant when combined with other factors. Thus, the interaction between cultivation methods and the proportion of native flour affected parameters such as protein and total digestible starch content. Based on the results obtained, breads made with 100% flour from traditional local wheat cultivars grown organically showed high levels of protein and complex carbohydrates, important nutrients in the human diet [50], which confirms the idea that traditional local wheat varieties are highly adaptable to organic production conditions [37]. Therefore, the recovery, cultivation and promotion of local cultivars are essential for developing crop varieties suited to organic farming and ensuring optimal nutritional quality in wheat-based products such as bread.

Although the present study was centered on the nutritional composition of Galician bread, other quality parameters such as texture, aroma and overall acceptability also play a key role in consumer perception. In particular, Galician bread is traditionally valued not only for its nutritional profile but also for its characteristic thick crust, moist crumb and intense cereal flavor. These attributes are believed to be influenced by the type and proportion of flour used. Therefore, future research should aim to include sensory and instrumental texture analyses in order to provide a more complete understanding of how local wheat varieties contribute to the overall quality of Galician bread.

Additionally, one limitation of this study is the use of duplicates in some analyses, which may reduce statistical power in detecting minor differences. However, the use of independent batches and a consistent factorial design provided robustness for evaluating the effects of variety, cultivation system and flour proportion.

## 5. Conclusions

This study highlights that the nutritional quality of Galician bread can be significantly improved through the use of local wheat varieties and higher proportions of local flour. Rather than acting independently, the factors evaluated (wheat variety, cultivation system and flour proportion) interacted in complex ways to shape the nutritional profile of the bread.

Bread made with 100% local varieties, ‘Caaveiro’ and ‘Callobre’, showed higher levels of protein, carbohydrates and essential minerals, which are fundamental for the optimal functioning of the human body. The local wheat variety also demonstrated an important influence on the bread composition, with each variety excelling in different nutritional aspects, such as protein for ‘Caaveiro’ and carbohydrate for ‘Callobre’. Although the cultivation system alone (organic vs. conventional) had a more limited effect, it became more relevant when combined with the highest proportion of local flour. Beyond individual effects, several interactions were identified. The combination of cultivation method, wheat variety and flour proportion influenced the nutritional profile in complex ways.

Overall, based on the results obtained, promoting organic farming and the recovery of traditional wheat varieties can enhance the nutritional value of Galician bread, supporting both environmental sustainability and local agricultural traditions. However, further studies are needed to confirm these potential benefits.

## Figures and Tables

**Table 1:** Wheat flour samples used in the Galician bread baking process obtained from two cultivation systems.

Local Variety	Percentage of Local Flour	Production System
‘Caaveiro’	100%	Organic
‘Caaveiro’	100%	Conventional
‘Caaveiro’	25%	Organic
‘Caaveiro’	25%	Conventional
‘Callobre’	100%	Organic
‘Callobre’	100%	Conventional
‘Callobre’	25%	Organic
‘Callobre’	25%	Conventional

**Table 2:** Nutritional profile of different Galician bread types analyzed made with 100% local wheat (‘Caaveiro’ and ‘Callobre’) flour and blends with 25% local wheat and 75% commercial flour.

Proportion and Variety	100% ‘Caaveiro’		100% ‘Callobre’		25% ‘Caaveiro’		25% ‘Callobre’	
Cultivation System	Organic	Conventional	Organic	Conventional	Organic	Conventional	Organic	Conventional
Moisture (g/100 g)	30.63 ± 3.30 ^b^	30.28 ± 2.89 ^b^	26.08 ± 3.90 ^b^	28.08 ± 2.47 ^b^	38.64 ± 1.74 ^a^	38.53 ± 2.02 ^a^	38.45 ± 1.59 ^a^	36.23 ± 4.62 ^a^
Protein (g/100 g)	13.21 ± 0.01 ^a^	11.86 ± 0.03 ^b^	10.66 ± 0.08 ^c^	10.36 ± 0.00 ^d^	9.30 ± 0.03 ^e^	9.18 ± 0.00 ^f^	9.30 ± 0.00 ^f^	9.97 ± 0.03 ^f^
Ash (g/100 g)	2.22 ± 0.08 ^a^	1.95 ± 0.02 ^a^	1.88 ± 0.02 ^a^	3.76 ± 2.69 ^a^	1.81 ± 0.07 ^a^	1.80 ± 0.11 ^a^	1.79 ± 0.02 ^a^	1.54 ± 0.02 ^a^
Fat (g/100 g)	1.22 ± 0.07 ^a^	1.09 ± 0.00 ^ab^	1.07 ± 0.08 ^ab^	1.11 ± 0.11 ^ab^	0.85 ± 0.10 ^bc^	0.63 ± 0.01 ^c^	0.67 ± 0.06 ^c^	0.68 ± 0.07 ^c^
CHO (g/100 g)	49.10 ± 2.07 ^b^	53.06 ± 0.47 ^ab^	60.98 ± 0.28 ^a^	57.45 ± 6.05 ^ab^	49.93 ± 3.83 ^ab^	49.27 ± 2.40 ^c^	49.89 ± 1.09 ^ab^	48.09 ± 3.03 ^b^
TS (g/100 g)	37.51 ± 0.36 ^cd^	39.58 ± 0.29 ^b^	42.08 ± 0.47 ^a^	38.10 ± 0.46 ^bcd^	34.80 ± 0.37 ^e^	35.35 ± 0.11 ^e^	36.52 ± 0.74 ^de^	39.12 ± 0.49 ^bc^
RS (g/100 g)	3.08 ± 0.80 ^a^	3.35 ± 1.28 ^a^	3.83 ± 1.11 ^a^	4.16 ± 1.56 ^a^	3.75 ± 0.07 ^a^	3.39 ± 1.10 ^a^	4.21 ± 0.39 ^a^	3.40 ± 0.69 ^a^
TDS (g/100 g)	34.43 ± 0.45 ^abc^	36.23 ± 1.57 ^ab^	38.25 ± 1.57 ^a^	33.94 ± 1.10 ^bc^	31.04 ± 0.30 ^c^	31.97 ± 1.21 ^bc^	32.31 ± 0.35 ^bc^	35.72 ± 1.18 ^ab^
RDS (g/100 g)	26.90 ± 5.94 ^a^	26.76 ± 6.30 ^a^	28.31 ± 5.25 ^a^	26.70 ± 6.62 ^a^	24.03 ± 3.77 ^a^	23.84 ± 5.13 ^a^	23.88 ± 4.92 ^a^	26.94 ± 5.13 ^a^
SDS (g/100 g)	0.72 ± 0.94 ^a^	1.52 ± 0.19 ^a^	0.98 ± 0.60 ^a^	0.60 ± 0.79 ^a^	0.68 ± 0.73 ^a^	1.33 ± 0.44 ^a^	0.92 ± 0.29 ^a^	0.80 ± 0.01 ^a^
Glucose (g/100 g)	0.01 ± 0.00 ^c^	0.01 ± 0.00 ^bc^	0.04 ± 0.01 ^abc^	0.06 ± 0.00 ^a^	0.04 ± 0.00 ^abc^	0.04 ± 0.03 ^abc^	0.04 ± 0.03 ^abc^	0.05 ± 0.00 ^ab^
Fructose (g/100 g)	0.01 ± 0.00 ^c^	0.03 ± 0.00 ^c^	0.09 ± 0.02 ^b^	0.13 ± 0.01 ^ab^	0.13 ± 0.01 ^ab^	0.13 ± 0.03 ^ab^	0.14 ± 0.03 ^ab^	0.14 ± 0.00 ^a^
Sucrose (g/100 g)	0.004 ± 0.00 ^ab^	0.004 ± 0.00 ^ab^	0.004 ± 0.00 ^ab^	0.007 ± 0.00 ^a^	0.003 ± 0.00 ^b^	0.003 ± 0.00 ^b^	0.006 + 0.00 ^ab^	0.004 ± 0.00 ^b^
Maltose (g/100 g)	0.61 ± 0.04 ^d^	0.47 ± 0.03 ^e^	0.34 ± 0.03 ^f^	0.25 ± 0.02 ^f^	1.07 ± 0.04 ^a^	1.04 ± 0.02 ^ab^	0.97 ± 0.04 ^b^	0.87 ± 0.02 ^c^
Fiber (g/100 g)	3.00 ± 1.88 ^a^	1.47 ± 0.01 ^a^	2.44 ± 1.90 ^a^	2.34 ± 0.17 ^a^	2.83 ± 0.25 ^a^	2.09 ± 0.10 ^a^	2.17 ± 0.26 ^a^	1.60 ± 0.07 ^a^
Amylose (g/100 g)	15.31 ± 0.12 ^cd^	14.78 ± 0.00 ^d^	15.28 ± 0.39 ^cd^	16.01 ± 0.99 ^bcd^	17.25 ± 0.20 ^ab^	16.68 ± 0.23 ^bc^	17.49 ± 0.03 ^ab^	18.51 ± 0.32 ^a^
AmyP(g/100 g)	84.69 ± 0.12 ^ab^	85.22 ± 0.00 ^a^	84.73 ± 0.39 ^ab^	83.99 ± 0.99 ^abc^	82.75 ± 0.20 ^cd^	83.33 ± 0.23 ^bc^	82.51 ± 0.03 ^cd^	81.49 ± 0.32 ^d^
Na (mg/100 g)	569.75 ± 31.40 ^a^	550.59 ± 14.73 ^a^	575.01 ± 8.02 ^a^	504.13 ± 20.25 ^b^	503.19 ± 29.74 ^b^	506.48 ± 8.60 ^b^	503.11 ± 18.30 ^b^	434.40 ± 18.60 ^c^
Mg (mg/100 g)	31.60 ± 1.09 ^d^	36.47 ± 0.96 ^c^	39.85 ± 0.59 ^b^	42.46 ± 1.70 ^a^	28.39 ± 1.40 ^e^	28.08 ± 0.68 ^e^	28.67 ± 1.41 ^e^	27.90 ± 1.10 ^e^
P (mg/100 g)	154.02 ± 7.35 ^c^	165.26 ± 3.34 ^b^	188.58 ± 4.17 ^a^	192.33 ± 6.75 ^a^	123.60 ± 6.25 ^d^	122.73 ± 2.32 ^d^	127.64 ± 4.54 ^d^	121.50 ± 4.66 ^d^
K (mg/100 g)	132.07 ± 3.60 ^c^	144.55 ± 1.75 ^b^	157.10 ± 2.87 ^a^	149.13 ± 4.67 ^b^	115.57 ± 5.80 ^d^	114.06 ± 1.67 ^d^	128.66 ± 5.09 ^c^	114.77 ± 4.69 ^d^
Ca (mg/100 g)	13.75 ± 0.81 ^e^	20.29 ± 1.15 ^bc^	28.72 ± 2.35 ^a^	21.72 ± 1.11 ^b^	21.17 ± 0.82 ^bc^	19.79 ± 0.89 ^bc^	18.48 ± 0.99 ^d^	19.05 ± 0.40 ^cd^
Mn (mg/100 g)	1.44 ± 0.02 ^c^	1.18 ± 0.03 ^d^	1.80 ± 0.02 ^b^	1.95 ± 0.08 ^a^	0.82 ± 0.04 ^e^	0.83 ± 0.01 ^e^	0.76 ± 0.04 ^e^	0.72 ± 0.03 ^f^
Fe (mg/100 g)	1.67 ± 0.43 ^cd^	2.85 ± 0.42 ^a^	1.99 ± 0.42 ^bc^	2.17 ± 0.23 ^b^	1.35 ± 0.09 ^d^	1.77 ± 0.20 ^bcd^	1.70 ± 0.20 ^cd^	1.72 ± 0.09 ^bcd^
Cu (mg/100 g)	0.27 ± 0.01 ^c^	0.39 ± 0.01 ^b^	0.40 ± 0.01 ^b^	0.44 ± 0.02 ^a^	0.18 ± 0.01 ^d^	0.19 ± 0.00 ^d^	0.17 ± 0.01 ^d^	0.18 ± 0.01 ^d^
Zn (mg/100 g)	1.26 ± 0.06 ^c^	1.23 ± 0.01 ^c^	1.66 ± 0.03 ^b^	1.80 ± 0.09 ^a^	0.91 ± 0.04 ^de^	1.00 ± 0.11 ^d^	0.85 ± 0.04 ^e^	0.86 ± 0.02 ^e^
Se (mg/100 g)	0.01 ± 0.00 ^a^	0.01 ± 0.00 ^a^	0.01 ± 0.00 ^a^	0.01 ± 0.00 ^a^	0.01 ± 0.00 ^a^	0.01 ± 0.00 ^a^	0.01 ± 0.00 ^a^	0.01 ± 0.00 ^a^

AmyP: amylopectin; CHO: carbohydrates; RDS: rapidly digestible starch; RS: resistant starch; SDS: slowly digestible starch; TDS: total digestible starch. Different letters indicate statistically significant differences according to a post hoc Tukey’s test.

**Table 3:** F-statistic and *p*-value obtained for the ANOVA (individual factors, 2-way factor interaction and 3-way factor interaction) of nutritional profile of all analyzed breads made with 100% local wheat (‘Caaveiro’ and ‘Callobre’) flour and blends with 25% local wheat and 75% commercial flour.

	Cultivation		Proportion		Variety		Cultivation × Proportion		Cultivation × Variety		Proportion × Variety		Cultivation × Proportion × Variety	
Variable	F	*p*-Value	F	*p*-Value	F	*p*-Value	F	*p*-Value	F	*p*-Value	F	*p*-Value	F	*p*-Value
Moisture	0.03	0.873	126.10	**0.000**	8.37	**0.006**	1.43	0.239	0.01	0.921	1.64	0.206	1.86	0.176
Protein	279.64	**0.000**	16554.02	**0.000**	2529.85	**0.000**	1159.65	**0.000**	810.10	**0.000**	5602.80	**0.000**	15.32	**0.004**
Ash	0.51	0.496	2.30	0.168	0.39	0.548	0.97	0.354	1.01	0.345	0.85	0.385	1.57	0.246
Fat	4.85	0.059	136.56	**0.000**	3.03	0.120	0.69	0.430	8.41	**0.020**	0.00	0.972	0.13	0.726
Carbohydrates	0.12	0.743	15.22	**0.005**	6.30	0.036	0.23	0.642	2.07	0.188	8.45	**0.019**	1.13	0.320
TS	1.96	0.199	166.54	**0.000**	93.03	**0.000**	32.40	**0.000**	20.26	**0.002**	7.30	**0.027**	82.60	**0.000**
RS	0.09	0.779	0.03	0.876	1.06	0.334	0.81	0.393	0.04	0.847	0.31	0.596	0.06	0.807
TDS	0.70	0.427	29.64	**0.001**	9.14	**0.016**	9.98	**0.013**	2.78	0.134	2.59	0.146	15.70	**0.004**
RDS	0.01	0.921	0.84	0.387	0.16	0.703	0.18	0.683	0.03	0.874	0.02	0.887	0.19	0.677
SDS	0.65	0.445	0.01	0.944	0.68	0.435	0.01	0.923	2.79	0.133	0.10	0.763	0.12	0.738
Glucose	3.27	0.089	5.54	**0.032**	13.09	**0.002**	0.12	0.739	1.31	0.268	7.63	**0.014**	0.24	0.629
Fructose	7.17	**0.016**	123.36	**0.000**	62.40	**0.000**	2.46	0.136	0.45	0.514	43.21	**0.000**	0.18	0.677
Sucrose	0.01	0.936	3.98	0.063	12.89	**0.002**	4.04	**0.062**	0.01	0.911	0.10	0.759	7.99	**0.012**
Maltose	47.96	**0.000**	2097.80	**0.000**	230.28	**0.000**	3.95	**0.064**	0.04	0.847	18.20	**0.000**	5.94	**0.027**
Fiber	2.19	0.170	0.10	0.756	0.18	0.681	0.04	0.838	0.01	0.912	0.42	0.531	0.00	0.960
Amylose	0.63	0.449	108.12	**0.000**	15.75	**0.004**	0.10	0.762	11.94	**0.009**	1.12	0.320	0.16	0.703
Amylopectin	0.63	0.449	108.12	**0.000**	15.75	**0.004**	0.10	0.762	11.94	**0.009**	1.12	0.320	0.16	0.703
Na	43.75	**0.000**	115.23	**0.000**	23.26	**0.000**	1.09	0.302	27.71	**0.000**	1.74	0.195	0.75	0.393
Mg	22.39	**0.000**	762.31	**0.000**	112.57	**0.000**	40.12	**0.000**	4.05	**0.051**	109.51	**0.000**	1.75	0.193
P	1.78	0.189	1170.49	**0.000**	115.98	**0.000**	13.52	**0.001**	4.55	**0.039**	96.63	**0.000**	0.14	0.712
K	5.47	0.024	554.96	**0.000**	86.80	**0.000**	18.23	**0.000**	49.66	**0.000**	11.52	**0.002**	3.00	0.091
Ca	0.87	0.357	18.98	**0.000**	88.71	**0.000**	0.07	0.800	70.81	**0.000**	207.51	**0.000**	126.70	**0.000**
Mn	8.32	**0.006**	5085.21	**0.000**	451.09	**0.000**	2.76	0.104	61.12	**0.000**	815.30	**0.000**	99.23	**0.000**
Fe	37.70	**0.000**	52.73	**0.000**	0.02	0.887	9.60	**0.004**	22.58	**0.000**	4.86	**0.033**	4.16	0.048
Cu	265.99	**0.000**	5580.89	**0.000**	208.75	**0.000**	205.33	**0.000**	59.08	**0.000**	311.45	**0.000**	75.75	**0.000**
Zn	9.28	**0.004**	1150.90	**0.000**	128.29	**0.000**	0.03	0.873	1.81	0.187	291.05	**0.000**	14.38	**0.000**
Se	0.06	0.816	0.02	0.884	2.09	0.156	0.05	0.831	1.05	0.312	0.04	0.849	0.10	0.754

F: F-statistic; RDS: rapidly digestible starch; RS: resistant starch; SDS: slowly digestible starch; TDS: total digestible starch. *p* < 0.05 were considered statistically significant. Bold values denote statistically significant differences.

## Data Availability

The original contributions presented in the study are included in the article, further inquiries can be directed to the corresponding author.

## References

[1] Arranz-Otaegui A., Gonzalez Carretero L., Ramsey M.N., Fuller D.Q., Richter T. (2018). Archaeobotanical evidence reveals the origins of bread 14,400 years ago in northeastern Jordan. Proc. Natl. Acad. Sci. USA.

[2] Luengo E., Pastor J., Saldaña G. (2023). Traditional breads from Spain. Traditional European Breads.

[3] MAPA Informe del Consumo de Alimentación en España 2023. https://www.mapa.gob.es/es/alimentacion/temas/consumo-tendencias/informe_2023_baja_tcm30-685878.pdf.

[4] European Commission (2019). Commission Regulation (EU) 2019/2182 of 16 December 2019 entering a name in the register of protected designations of origin and protected geographical indications [Pan Galego (PGI)]. Off. J. Eur. Union.

[5] Estévez-López R.D., García-Gómez B., Vázquez-Odériz M.L., Ferreiro N.M., Romero-Rodríguez M.Á. (2021). Influence of bread shape on the sensory characteristics of Galician breads: Development of lexicon, efficacy control of the trained panel and establishment of a sensory profile. LWT.

[6] García-Gómez B., Fernández-Canto N., Vázquez-Odériz M.L., Quiroga-García M., Muñoz-Ferreiro N., Romero-Rodríguez M.Á. (2022). Sensory descriptive analysis and hedonic consumer test for Galician type breads. Food Control.

[7] Guzmán C., Ibba M.I., Álvarez J.B., Sissons M., Morris C. (2022). Wheat quality. Wheat Improvement.

[8] Aghalari Z., Dahms H.-U., Sillanpää M. (2022). Evaluation of nutrients in bread: A systematic review. J. Health Popul. Nutr..

[9] Păucean A., Șerban L.-R., Chiș M.S., Mureșan V., Pușcaș A., Man S.M., Pop C.R., Socaci S.A., Igual M., Ranga F. (2024). Nutritional composition, in vitro carbohydrates digestibility, textural and sensory characteristics of bread as affected by ancient wheat flour type and sourdough fermentation time. Food Chem. X.

[10] Urquijo L., Romero Rodríguez M., Pereira Lorenzo S. (2018). ¿Cómo recuperar los ecotipos autóctonos?. Respostas ás Preguntas Sobre o Pan e o Cereal do País.

[11] Arzani A., Ashraf M. (2017). Cultivated ancient wheats (*Triticum* spp.): A potential source of health-beneficial food products. Comprehensive Rev. Food Sci. Food Saf..

[12] Adhikari S., Kumari J., Jacob S.R., Prasad P., Gangwar O.P., Lata C., Thakur R., Singh A.K., Bansal R., Kumar S. (2022). Landraces—Potential treasure for sustainable wheat improvement. Genet. Resour. Crop Evol..

[13] Zamaratskaia G., Gerhardt K., Wendin K. (2021). Biochemical characteristics and potential applications of ancient cereals—An underexploited opportunity for sustainable production and consumption. Trends Food Sci. Technol..

[14] Vindras-Fouillet C., Goldringer I., van Frank G., Dewalque M., Colin A., Montaz H., Berthellot J.-F., Baltassat R., Dalmasso C. (2021). Sensory analyses and nutritional qualities of wheat population varieties developed by participatory breeding. Agronomy.

[15] Sumberg J., Giller K.E. (2022). What is ‘conventional’ agriculture?. Glob. Food Secur..

[16] Câmara-Salim I., Almeida-García F., Feijoo G., Moreira M.T., González-García S. (2021). Environmental consequences of wheat-based crop rotation in potato farming systems in Galicia, Spain. J. Environ. Manag..

[17] Rebolledo-Leiva R., Almeida-García F., Pereira-Lorenzo S., Ruíz-Nogueira B., Moreira M.T., González-García S. (2022). Determining the environmental and economic implications of lupin cultivation in wheat-based organic rotation systems in Galicia, Spain. Sci. Total Environ..

[18] Wang J., Chatzidimitriou E., Wood L., Hasanalieva G., Markellou E., Iversen P.O., Seal C., Baranski M., Vigar V., Ernst L. (2020). Effect of wheat species (*Triticum aestivum* vs *T. spelta*), farming system (organic vs conventional) and flour type (wholegrain vs white) on composition of wheat flour. Food Chem. X.

[19] Pontonio E., Arora K., Dingeo C., Carafa I., Celano G., Scarpino V., Genot B., Gobbetti M., Di Cagno R. (2021). Commercial organic versus conventional whole rye and wheat flours for making sourdough bread: Safety, nutritional, and sensory implications. Front. Microbiol..

[20] Stern A.L., Berstein J., Jones S.S., Blumberg J.B., Griffin T.S. (2021). The impacts of germinating organic wheat: Effects on phytic acid, resistant starch, and functional properties of flour, and sensory attributes of sourdough bread. Int. J. Food Sci. Technol..

[21] Fernández-Canto N., García-Gómez M.B., Vázquez-Odériz M.L., Lombardero-Fernández M., Pereira-Lorenzo S., Cobos Á., Díaz O., Romero-Rodríguez M.Á. (2024). Autochthonous Wheat Grown in Organic and Conventional Systems: Nutritional Quality of Flour and Bread. Foods.

[22] Fernández-Canto M.N., García-Gómez M.B., Boado-Crego S., Vázquez-Odériz M.L., Muñoz-Ferreiro M.N., Lombardero-Fernández M., Pereira-Lorenzo S., Romero-Rodríguez M.Á. (2022). Element Content in Different Wheat Flours and Bread Varieties. Foods.

[23] AOAC (2019). Official Methods of Analysis of Association of Official Analytical Chemists International.

[24] R Core Team (2021). R: A Language and Environment for Statistical Computing.

[25] Lê S., Josse J., Husson F. (2008). FactoMineR: An R Package for Multivariate Analysis. J. Stat. Softw..

[26] Bhatt C.M., Nagaraju J. (2010). Studies on Electrical Properties of Wheat Bread as a Function of Moisture Content during Storage. Sens. Instrum. Food Qual. Saf..

[27] Carocho M., Morales P., Ciudad-Mulero M., Fernández-Ruiz V., Ferreira E., Heleno S., Rodrigues P., Barros L., Ferreira I.C.F.R. (2020). Comparison of Different Bread Types: Chemical and Physical Parameters. Food Chem..

[28] Pico J., Martínez M.M., Martín M.T., Gómez M. (2015). Quantification of Sugars in Wheat Flours with an HPAEC-PAD Method. Food Chem..

[29] Boukid F., Vittadini E., Prandi B., Mattarozzi M., Marchini M., Sforza S., Sayar R., Seo Y.W., Yacoubi I., Mejri M. (2018). Insights into a Century of Breeding of Durum Wheat in Tunisia: The Properties of Flours and Starches Isolated from Landraces, Old and Modern Genotypes. LWT.

[30] Calvin O. (2016). Starch and Modified Starch in Bread Making: A Review. Afr. J. Food Sci..

[31] AESAN Informe del Comité Científico de La Agencia Española de Seguridad Alimentaria y Nutrición (AESAN) de Revisión y Actualización de Las Recomendaciones Dietéticas Para La Población Española. https://www.aesan.gob.es/AECOSAN/docs/documentos/seguridad_alimentaria/evaluacion_riesgos/informes_comite/RECOMENDACIONES_DIETETICAS.pdf.

[32] Sitrin M.D. (2014). Digestion and Absorption of Carbohydrates and Proteins. The Gastrointestinal System.

[33] Bulut S. (2022). Mineral Content of Some Bread Wheat Cultivars. Cereal Res. Commun..

[34] European Commission Council Directive of 24 September 1990 on Nutrition Labelling for Foodstuffs. https://eur-lex.europa.eu/LexUriServ/LexUriServ.do?uri=CONSLEG:1990L0496:20081211:EN:PDF.

[35] Lockyer S., Spiro A. (2020). The Role of Bread in the UK Diet: An Update. Nutr. Bull..

[36] Valli V., Taccari A., Di Nunzio M., Danesi F., Bordoni A. (2018). Health Benefits of Ancient Grains. Comparison among Bread Made with Ancient, Heritage and Modern Grain Flours in Human Cultured Cells. Food Res. Int..

[37] Lammerts van Bueren E.T., Jones S.S., Tamm L., Murphy K.M., Myers J.R., Leifert C., Messmer M.M. (2011). The Need to Breed Crop Varieties Suitable for Organic Farming, Using Wheat, Tomato and Broccoli as Examples: A Review. NJAS Wageningen J. Life Sci..

[38] Boukid F., Gentilucci V., Vittadini E., De Montis A., Rosta R., Bosi S., Dinelli G., Carini E. (2020). Rediscovering Bread Quality of “Old” Italian Wheat (*Triticum aestivum* L. ssp. *aestivum*) through an Integrated Approach: Physicochemical Evaluation and Consumers’ Perception. LWT.

[39] Melini V., Melini F., Acquistucci R. (2021). Nutritional Characterization of an Italian Traditional Bread from Ancient Grains: The Case Study of the Durum Wheat Bread “Pane Di Monreale”. Eur. Food Res. Technol..

[40] Lamlom S.F., Irshad A., Mosa W.F.A. (2023). The Biological and Biochemical Composition of Wheat (*Triticum aestivum*) as Affected by the Bio and Organic Fertilizers. BMC Plant Biol..

[41] EL-Guibali A. (2016). Effect of Organic and Mineral Fertilization on Wheat Yield and Quality. J. Soil Sci. Agric. Eng..

[42] Nasiroleslami E., Mozafari H., Sadeghi-Shoae M., Habibi D., Sani B. (2021). Changes in Yield, Protein, Minerals, and Fatty Acid Profile of Wheat (*Triticum aestivum* L.) under Fertilizer Management Involving Application of Nitrogen, Humic Acid, and Seaweed Extract. J. Soil Sci. Plant Nutr..

[43] Vrček I.V., Čepo D.V., Rašić D., Peraica M., Žuntar I., Bojić M., Mendaš G., Medić-Šarić M. (2014). A Comparison of the Nutritional Value and Food Safety of Organically and Conventionally Produced Wheat Flours. Food Chem..

[44] Fernández-Canto N., García-Gómez M.B., Vázquez-Odériz M.L., Lombardero-Fernández M., Pereira-Lorenzo S., Cobos Á., Díaz O., Vázquez M., Romero-Rodríguez M.Á. (2024). Impact of Organic and Conventional Farming Practices on the Multidimensional Characteristics of Flour and Indirectly on Bread. LWT.

[45] Hellemans T., Landschoot S., Dewitte K., Van Bockstaele F., Vermeir P., Eeckhout M., Haesaert G. (2018). Impact of Crop Husbandry Practices and Environmental Conditions on Wheat Composition and Quality: A Review. J. Agric. Food Chem..

[46] Rozbicki J., Ceglińska A., Gozdowski D., Jakubczak M., Cacak-Pietrzak G., Mądry W., Golba J., Piechociński M., Sobczyński G., Studnicki M. (2015). Influence of the Cultivar, Environment and Management on the Grain Yield and Bread-Making Quality in Winter Wheat. J. Cereal Sci..

[47] Mazzoncini M., Antichi D., Silvestri N., Ciantelli G., Sgherri C. (2015). Organically vs Conventionally Grown Winter Wheat: Effects on Grain Yield, Technological Quality, and on Phenolic Composition and Antioxidant Properties of Bran and Refined Flour. Food Chem..

[48] Zörb C., Niehaus K., Barsch A., Betsche T., Langenkämper G. (2009). Levels of Compounds and Metabolites in Wheat Ears and Grains in Organic and Conventional Agriculture. J. Agric. Food Chem..

[49] Smith O.M., Cohen A.L., Rieser C.J., Davis A.G., Taylor J.M., Adesanya A.W., Jones M.S., Meier A.R., Reganold J.P., Orpet R.J. (2019). Organic Farming Provides Reliable Environmental Benefits but Increases Variability in Crop Yields: A Global Meta-Analysis. Front. Sustain. Food Syst..

[50] Shewry P.R., Hey S.J. (2015). The Contribution of Wheat to Human Diet and Health. Food Energy Secur..

